# Increased livestock weight gain from improved water quality in farm dams: A cost-benefit analysis

**DOI:** 10.1371/journal.pone.0256089

**Published:** 2021-08-16

**Authors:** Leo Dobes, Mason Crane, Tim Higgins, Albert I. J. M. Van Dijk, David B. Lindenmayer

**Affiliations:** 1 Sustainable Farms, College of Business and Economics, The Australian National University, Canberra, Australian Capital Territory, Australia; 2 Sustainable Farms, Fenner School of Environment and Society, The Australian National University, Canberra, Australian Capital Territory, Australia; International Centre for Integrated Mountain Development (ICIMOD), Kathmandu, Nepal, NEPAL

## Abstract

Access to water is a critical aspect of livestock production, although the relationship between livestock weight gain and water quality remains poorly understood. Previous work has shown that water quality of poorly managed farm dams can be improved by fencing and constructing hardened watering points to limit stock access to the dam, and revegetation to filter contaminant inflow. Here we use cattle weight gain data from three North American studies to develop a cost-benefit analysis for the renovation of farm dams to improve water quality and, in turn, promote cattle weight gain on farms in south-eastern Australia. Our analysis indicated a strong likelihood of positive results and suggested there may be substantial net economic benefit from renovating dams in poor condition to improve water quality. The average per-farm Benefit-Cost Ratios based on deterministic assumptions was 1.5 for New South Wales (NSW) and 3.0 for Victoria in areas where rainfall exceeds 600mm annually. Our analyses suggested that cattle on farms in NSW and Victoria would need to experience additional weight gain from switching to clean water of at least 6.5% and 1.8% per annum respectively, to break even in present value terms. Monte Carlo simulation based on conservative assumptions indicated that the probability of per-farm benefits exceeding costs was greater than 70%. We recommend localised experiments to assess the impact of improved water quality on livestock weight gain in Australian conditions to confirm these expectations empirically.

## 1. Introduction

The consumption of water and food by ruminants is closely linked, and access to drinking water is important for the associated consumption of food by domestic livestock such as cattle and sheep [1, 2]. This has motivated the construction of a large number of artificial water points in agricultural landscapes worldwide [e.g. 3–6]. For example, the Murray-Darling Basin, the most important food production region in Australia, supports more than 650 000 farm dams that provide domestic livestock access to drinking water [5]. Continent-wide, there is an estimated 1.765m farm dams in Australia [7].

Only three studies—all from North America—have explored empirical relationships between water quality and weight gain in beef cattle. Two of these, Willms, Kenzie [8] and Lardner, Kirychuk [9], suggest that an adequate supply of good quality water may be important for improving livestock health and productivity, and hence farm profitability, but Crawford, Cole [10] detected no increase in weight due to cleaner water.

**Fig 1:**
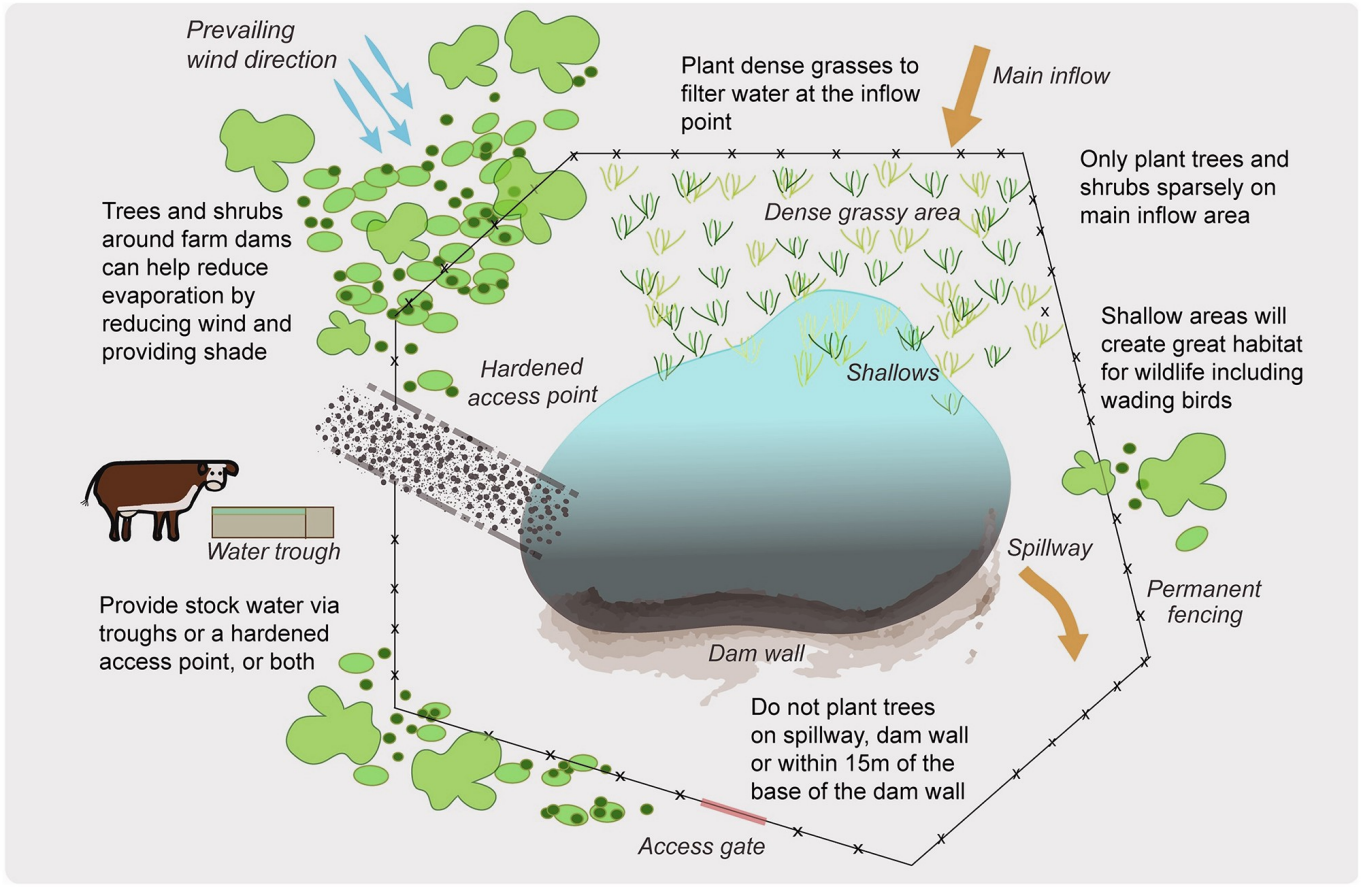
Management interventions aimed at improving water quality in farm dams in south-eastern Australia (http://www.sustainablefarms.org.au/research).

In south-eastern Australia, many farm dams appear to be in relatively poor condition [6], are often suffused with faecal and other organic matter and characterised by poor quality water. Recent work in south-eastern Australian agricultural regions (https://www.sustainablefarms.org.au/about) has sought to quantify the effects of management interventions on improving the condition of farm dams and the water they contain [see Fig 1; 11]. For example, fences are established to preclude access by livestock to farm dams to limit the direct deposit of faecal matter and urine to the water body. Fencing has also been found to reduce degradation of dam walls and the margins of dams, and promote the establishment of native vegetation to help filter contaminants from inflows to dams from surrounding grazed pastures. Preliminary work from replicated cross-sectional studies [*sensu* 12] has shown that relative to unmanaged farm dams, those where there have been management interventions such as fencing to limit livestock access were characterised by significantly lower levels of *E*.*coli* and thermotolerant coliforms as well as lower levels of turbidity, nitrogen and phosphorus [11]. Importantly, an overarching aim of restoration efforts around farm dams is to bring water quality measures close to, or below, quality thresholds set for animal consumption [13]. Westgate, Crane [11] also found that compared to open access dams, managed farm dams support higher levels of native macroinvertebrate species richness and abundance including many species considered to be indicators of high water quality.

The renovation of dams in the ways depicted in Fig 1 requires labour, fencing materials, establishment of tree and ground cover, and the construction of hardened access points or reticulation to troughs. It is therefore pertinent to quantify whether the resource costs involved justify the postulated economic benefits, and in particular, the increased weight gain of livestock due to more effective metabolism and greater feed intake. We report the results of a cost-benefit analysis of renovating farm dams in northern Victoria and south-eastern New South Wales, Australia. We focused on areas where the annual rainfall exceeded 600mm in the southern part of the Grassy Box-Gum woodlands of south-eastern Australia. This was because such areas are more likely to support farms focussed primarily on cattle production. We draw on data on increased growth rates of cattle drinking clean rather than poor quality water (as reported by studies in North America; [see 8–10]), coupled with data on infrastructure and labour costs and cattle sales and prices. Our analyses produced evidence of strong positive net results. The uncertainty associated with the variables used to estimate economic benefits—particularly, the assumed enhancement of growth rates for cattle in an Australian environment—led us to also apply Monte Carlo Analysis to assess the likelihood of the positive results obtained.

## 2. Background—Water quality and weight gain in cattle

Few studies have quantified differences in weight gain between livestock consuming good and poor quality water. Willms, Kenzie [8, Table 3, p. 457] found 23% greater weight gain for steers drinking clean water from a trough, compared to those drinking directly from open access dams in southern Alberta and British Columbia. Lardner, Kirychuk [9, Table 2, p. 101] reported an average annual 9% weight gain for steers in Saskatchewan, but did not compare the effect of cattle-contaminated water with naturally clean water from a spring, well, or river. Conversely, Crawford, Cole [10, p. 4] concluded that ‘*water source had no effect on water intake or rate of [weight] gain’* in a study in Missouri.

We searched the literature for any studies that quantify relationships between water quality and weight gain in domestic livestock in Australia, but failed to identify any studies conducted in Australia for cattle or sheep. The journals searched included *Rural Research*: *a CSIRO quarterly*, 1952–1997; *Animal Production Science* [formerly known as the *Australian Journal of Experimental Agriculture and Animal Husbandry]*, 1961–2019; *Australian Veterinary Journal*, 1920–2019, and the *Australian Journal of Agricultural and Resource Economics*, 1959–2019, and the websites of Land and Water Australia, CSIRO, and Meat and Livestock Australia. Notably, interviews with over 20 veterinarians, government agricultural officials, academics, farmers and researchers (see Appendix I in S1 File), also failed to uncover any additional peer-reviewed scientific information on relationships between water quality and weight gain in domestic livestock in Australia. In Table 1, we summarise publicly available data about differences in live-weight gain by steers (castrated male cattle) associated with differences in the quality of the water consumed from dams.

**Table 1:** Weight gain by steers drinking poor versus good quality water (kg/day).

Water source	Crawford et al.	Willms et al.	Lardner et al.
	(1997, Table 2)	(2002, Table 3)	(2005, Table 2)
Cattle have direct access to a dam	0.46	0.64	0.97
Water pumped into trough from fenced dam		0.66	1.00
Well, spring or river water in trough	0.46	0.79	
Untreated dam water aerated continuously, pumped into trough			1.06
Untreated dam water coagulated & chlorinated, pumped into trough			1.05

There are some complexities associated with water quality and weight gain in domestic livestock. For example, sodium chloride can be toxic to livestock at certain levels [14], but, at lower levels, can encourage increased drinking, and, in turn, increased consumption of feed and therefore greater weight gain [15, 16]. A further complication is that it is difficult (and possibly impossible) to define a unique unit of measurement for water quality, although Total Dissolved Salts is sometimes used as a non-specific indicator [15, 17].

We acknowledge that other factors can influence weight gain in cattle in addition to access to high-quality water, including livestock breed and genetic variation [18], conjoint grazing of cattle and sheep [19], restricted water intake [20], the temperature of drinking water [21], pasture biomass, and feed supplements [22].

## 3. Cost-benefit analysis of renovating dams: A deterministic approach

The production approach we adopted aimed to quantify the benefit of additional weight gain due to consumption of good versus poor-quality water, compared to the costs of installing the requisite dam infrastructure. We did not model any specific positive or negative externalities, although increased biodiversity in renovated dams [23, 24], reduced greenhouse gas emissions [6] and mental health outcomes for farmers (Professor P. Batterham, Director, Centre for Mental Health Research, The Australian National University, personal communication, November 23 2020) are candidates for future analysis. Notably, we also did not account for periodic and acute livestock health issues associated with poor quality drinking water such as poisoning associated with blue green algae blooms or disease outbreaks such as Leptospirosis.

We set the period of analysis at 50 years to reflect the likely life of a well-maintained renovated dam built to a high standard. We used a 7% per annum real social discount rate because it has become standard practice among Australian Commonwealth and State Government agencies [25] and its harmonised use permits comparisons with alternative government projects.

### 3.1 Estimation of benefits

Additional weight gained by steers with access to clean drinking water was the main source of benefits attributed to improving the condition of farm dams. Its value was estimated as the product of the additional weight gain in a year, the per kilogram liveweight price of steers, and the number of beef cattle sold per average farm.

In the absence of evidence on relationships between water quality and weight gain under Australian conditions, we used the North American results reviewed in Section 2 above. A hypothesised 11%additional weight gain was used for the deterministic results presented in Table 3. This is the approximate arithmetic average weight gain found by the three North American studies, and our decision to use this figure is predicated on the assumption that the results of the three North American studies are equally credible. Farmers were assumed to breed their own cattle, and sell them as Feeder Steers (330 to 400 kilograms). Steers drinking water from a dam in good condition would gain 11% more weight (approximately 44 kilograms) than those drinking from a dam in poor condition if sold at the top of the weight range. We address uncertainty in weight gain in Section 4 using Monte Carlo simulation.

An alternative business model for livestock production is for a “restocker” farmer to forgo maintaining a dedicated herd; instead purchasing restocker steers each year and turning off all of them as feeder steers for finishing at a feedlot in about a year. This approach allows a larger number of saleable cattle to be accommodated on a property because bulls and cows with calves are excluded. We acknowledge that some farmers in NSW and Victoria may specialise as restockers, and others as breeders. However, it would be unrealistic to posit that all farmers in our project area act purely as restockers, because restocker steers could then be sourced each year only from outside our project area.

Sale prices for cattle exhibited a step-change in the period 2015–2019, compared with the previous five years [26]. The mean liveweight sale price in 2019 dollars of Feeder Steers was $A3.19 per kilogram in NSW for 2015–2019. The corresponding Victorian price was $A3.03.

We present data on the number of cattle sold by an average farm annually in Table 2. We used Local Government Areas (LGAs) with annual rainfall exceeding 600mm in the southern part of the Grassy Box-Gum woodlands of south-eastern Australia. This was because they are more likely to contain farms focussed primarily on cattle production. We assumed the value of the additional weight gain based on the average NSW farm selling 217 beef cattle annually. The equivalent figure used for Victorian farms was 177 beef cattle sold annually. However, the data in Table 2 are based on a sample of Australian farms collected as part of the Australian Agricultural and Grazing Industries Survey (AAGIS; (https://www.agriculture.gov.au/abares/research-topics/surveys/farm-survey-data) which excludes farms with a gross income (Estimated Value of Agricultural Operations) of less than $40,000 per annum. Table 2 thus excludes farms with herds of less than approximately 100 animals (Dale Ashton, Senior Economist, Farm Survey Analysis, ABARES, 3 April 2020, personal communication).

**Table 2:** Characteristics of NSW and Victorian beef cattle farms in the southern sheep-wheat belt of south-eastern Australia.

Financial years	2015	2016	2017	2018	mean
**New South Wales**					
Beef cattle sold per farm	257	222	159	228	217
Beef cattle at 30 June per farm	383	282	417	301	346
Area operated (ha) per farm	1672	1705	1208	1373	1490
Stocking rate (head/ha) per farm	0.24	0.18	0.33	0.24	0.25
Number of farms	598	605	349	414	492
**Victoria**					
Beef cattle sold per farm	145	235	138	188	177
Beef cattle at 30 June per farm	320	489	320	511	410
Area operated (ha) per farm	408	740	447	622	554
Stocking rate (head/ha) per farm	0.80	0.66	0.72	0.80	0.75
Number of farms	3029	1101	2076	1350	1889

***Source***: Data were provided by the Australian Bureau of Agricultural and Resource Economics and Sciences (ABARES) from its Australian Agricultural and Grazing Industries Survey (https://www.agriculture.gov.au/abares/research-topics/surveys/farm-survey-data), based on Local Government Areas with annual rainfall exceeding 600mm in the Sustainable Farms project area. ***Notes***: (a) Financial years: e.g. 2015 refers to 2014–15. (b) Local Government Areas included for NSW: Albury, Bathurst Regional, Blayney, Cabonne, Cootamundra-Gundagai regional, Goulburn Mulwaree, Greater Hume, Oberon, Orange, Queanbeyan-Palerang regional, Snowy Monaro region, Snowy Valleys, Upper Lachlan Shire, Wingecarribee, Yass Valley; and for Victoria: Alpine, Benalla, East Gippsland, Indigo, Mansfield, Murrindindi, Towong, Wangaratta, Wodonga.

A fenced dam will lead to dung and urine otherwise deposited in the dam to be distributed elsewhere on the property instead. Based on results for dairy cows in Missouri [27] as reported by [28 p. 13] derived an annual estimate of fertiliser costs avoided of $616 USD (about $1047 AUD in 2019 prices) for a 100-cow herd. We have used that estimate in Table 3 for analytical completeness but were unable to attach a level of confidence to it.

**Table 3:** Deterministic analysis of per farm Net Present Value (NPV) of renovating dams on beef cattle farms in NSW and Victoria over a 50-year period. ($’000).

Item	NSW: per farm	Victoria: per farm
	PV($2019)	PV($2019)
***Benefits***		
Value of additional weight gain	420	326
Fertiliser saving	50	59
Saving due to reduced frequency of desilting dams	74	28
Present Value ($2019) of benefits	544	413
***Costs***		
Construction of dam fence	204	76
Construction of hardened watering point	114	42
Planting vegetation	14	5
Dam fence maintenance	13	5
Hardened watering point maintenance	27	10
Present Value ($2019) of costs	372	138
Net Present Value ($2019)	172	275
Benefit Cost Ratio	1.5	3.0

***Notes***: PV($2019): Present Value (PV) expressed in real (2019) dollars by converting all prices using the Consumer Price Index (CPI) published by the Australian Bureau of Statistics (Cat. No. 6401.0). Present values are based on a 50-year period and 7% per annum real discount rate. The farms included in the calculations are those in Local Government Areas of the Grassy Box-Gum woodlands used in Table 2. The higher Net Present Value (NPV) per farm in Victoria is due to the greater number of cattle per ha (an average of 0.74 for Victoria vs 0.23 cattle per ha for NSW).

An important feature of a renovated dam is the establishment of vegetation in the inflow area (Fig 1) to trap soil and other matter, reducing the frequency of desilting required. Desilting a 1600 square metre dam costs about $3,000. We assumed that a dam in poor condition would need to be desilted every ten years, and the present value of the cost would be $2,997 using a 7% per annum real discount rate, over 50 years at 2019 prices. If a renovated dam was cleaned at years 20, 40 and 60, the present value of the cost would be $1,027. To ensure comparability, we treated the difference of $144 in Equivalent Annual Value ($217 and $73 respectively) as an annuity over 50 years at a real discount rate of 7% per annum. We estimated the present value of the saving in desilting cost to be $1,986 per dam.

Incorporation of a hardened watering point in a renovated dam may reduce the incidence of cattle becoming bogged. However, information on the cost and frequency of drowning, or of animal extraction from a dam is limited. A survey of Queensland farmers by Sillar Associates [28, see p. 17] in the Mary River catchment considered the effect to be ‘*zero to 0*.*5% of overall herd mortality in dairy herds and to be negligible in beef herds’*. To maintain a conservative estimate of benefits, we assumed there were no savings from avoided bogging or drowning incidents.

Evidentiary Pty Ltd [29, p. 19] reported that ‘*research on water contaminants and their effects on cattle performance are sparse’*. Sillar Associates [28, p. 17] considered that the incidence of mastitis in dairy cattle would depend on the quality of water, as well as on other predisposing factors such as milking shed hygiene, but ‘*is generally not a problem in beef cattle’*. Therefore, we made no allowance for the avoided cost of infections in our analysis.

### 3.2 Resource costs of renovating farm dams

A major determinant of costs in our analysis was the assumed size and number of renovated dams on each farm. We assumed there would be one renovated dam for every 40 hectares (~ 100 acres) of pasture, with the dimensions of each dam being approximately 40x40 metres, surrounded by 400 metres of cattle-proof fencing. This assumption accords roughly with surface hydrology points identified in Geoscience Australia datasets [30] for Catchment Scale Land Use of Australia categories 4 (grazing on native pasture) and 5 (grazing on modified pasture) (Table 2). NSW farmers would have an average of 37.25 dams per farm and Victorian farmers would have 13.85 dams per farm in those Local Government Areas (LGAs) with annual rainfall above 600mm.

We obtained data on the cost of fencing and hardened watering points suitable for dams used by cattle from established agricultural equipment suppliers and contractors in the Gundagai district of NSW. We conservatively estimated fencing costs at $5475 for each dam: 400-m hinged joint fence for a high-pressure area includes $3285 (four plain wires, two barbed wires, galvanised steel posts every 3m and maxi-post every 5^th^ post, and a gate), plus $2190 in labour cost. These costs reflect an assumed high quality of dam fences, designed to require less maintenance. We conservatively estimated the Present Value of maintenance costs as $349.60 per dam, calculated as one day of labour (7 hours) at $50 per hour every ten years over a 50-year period.

We based construction costs for a hardened watering point for each dam on 18 cubic metres of gravel and cartage ($560), spreading gravel ($100), 40m extra fencing including labour ($1300), and 24m submersible fence including labour ($1088). A conservative estimate of maintenance costs was based on gravel and other materials including delivery ($300), machinery and operator ($200) every ten years, and submersible mesh at $400 ($300 material and 2 hours labour at $50 an hour) replaced every 15 years. To ensure an equal cycle of gravel and mesh maintenance, we estimated the present values over 60 years, with an Equivalent Annual Value (EAV) calculated as $52.14. The annuity over 50 years of the EAV yielded a rounded present value of $720.

We estimated the cost of planting vegetation around each dam at $374.30. We based this on estimated costs of $107.80 for 20 trees and 50 shrubs at $1.54 each (including tubestock, stakes and cartons), $28.50 for 30 grass and sedge seedlings at $0.95 each, and $238 for labour cost at $2.38 per plant (pers. comm. Kathie Le Busque, Project Officer, West Hume Landcare, September 20 2020).

We did not include excavation costs because it was assumed that farmers would, as far as possible, renovate existing dams. While this assumption will partially underestimate costs, it was deemed reasonable because existing dams are already situated in the most appropriate locations for harvesting water, and they already gather the requisite amount of water for the cattle on the farm. Immediate excavation of existing dams would also result in loss of the water they contain. Where deepening of dams is required, this action may be implemented during desilting, probably at a time when water levels are low.

The existence of a dam and the surrounding fenced area involves a loss of pasture. However, no use can be made of pasture land without a water source for cattle. It is at least arguable that the pasture space occupied by a dam has no alternative use on a cattle farm with no water, so we deemed its opportunity cost to be zero. Further, a survey of 218 Victorian landholders indicated that any loss of grazing land was not of concern to 76% of respondents undertaking riparian fencing projects, ‘*as it was relatively minor across the property as a whole and the overall gains more than offset the costs*’ [31, p. 32].

We present the results of the cost-benefit analysis in Section 5.

## 4. Incorporating uncertainty into the estimation of benefits and costs

Because of uncertainty associated with the variables introduced in Section 3, particularly the assumed additional growth rates for cattle, we conducted a Monte Carlo simulation to provide insight into a plausible range for the cost-benefit estimates. We simulated 100,000 hypothetical farms for a 50-year period and extracted data on quantiles for net benefits. Simulation results are given in Section 5. The assumptions underlying the Monte Carlo simulation are given in this section.

### 4.1 Percentage weight gain

A major limitation in our simulations is the sparsity of literature on the critical impact on weight gain. Only three studies were available (Table 1) from which to infer distributional information. We treated the results of the three North American studies as equally probable, but note that the values reported are themselves average percentage weight gains for the cattle, and that each is based on limited samples, specific locations with specific environmental conditions, and during specific time periods. Therefore, we extended the range of possible values of weight gain beyond the average values from the three studies by applying a uniform distribution across the range of 0% to 23% (i.e., the lower and upper average values reported), such that all values within this range are taken to be equally probable.

Due to the sparsity of available data, and uncertainty about the true weight gain and variability of weight gain in the Australian environment, we take a conservative approach. The annual percentage weight gain for each of the 100,000 simulated farms for the first period (t = 1) was sampled from the uniform distribution with bounds of 0% and 23%, and this sample annual percentage weight gain was assumed to persist for each farm for the duration of the simulation period. This method allows for the possibility of permanent idiosyncratic differences in weight gain between farms, due, for example, to perennial local environmental conditions, mineral content of soil lining of the dam, pasture composition and quality, and local livestock genetics.

A shortcoming of this method is that it does not explicitly allow for variability in weight gain for each simulated farm across different years, which might arise due to changes in environmental conditions (rainfall and abundance/quality of forage). For example, Lardner, Kirychuk [9] provide evidence that the effect of improved water quality on weight gain is less pronounced in years of poor rainfall. Although our simulations do not include *yearly* variability in rainfall, the simulated weight gains are based on average increased weight gain from the three Northern American studies, and these averages reflect the climatic conditions over the periods of study which include periods of low rainfall.

An alternative simulation approach would be to assume that each farm in the 50-year simulation experiences periods of high and low weight gain, reflecting variability in the uniform distribution. However, a shortcoming of this alternative approach is that each farm’s simulated *average* weight gain would be close to the average of the uniform distribution, and as a consequence the method would underestimate the true variability in Net Present Value (NPV) if idiosyncratic differences exist between farms. A preferred model may be one that allows for permanent idiosyncratic differences while still incorporating yearly variability, however, choosing parameter values for such a model is currently not feasible given the sparsity of available data.

### 4.2 Prices

We expressed nominal prices per kilogram for feeder steers from 2003 to 2019 for Victoria and NSW in 2019 dollars. Analysis of the data leads to the selection of the following model for price, which was then applied to simulate real prices per kilogram for Victoria:

$$Price{}_{t}=Price_{t-1}\times exp\left(r_{t}\right),$$

where *r*_*t*_ is the logarithm of real returns and is simulated from a normal distribution with mean of zero and standard deviation of 0.14, which is equal to the standard deviation in log real returns from the observed data between 2003 and 2019. The initial price was taken as $3.03 being the average Victorian price per kilogram between 2015 and 2019. We found an almost perfect correlation (Pearson’s correlation coefficient (r) = 99.8%) between prices for NSW and Victoria. NSW prices are consistently higher by an average of approximately $0.16 (between 2015 and 2019). Therefore, simulated prices per kilogram for NSW were taken as equal to the simulated prices for Victoria plus $0.16.

### 4.3 Other benefit components

There are limitations in choosing probability distributions for other assumptions in the model due to sparse data. For the number of cattle sold per farm, individual farm-level information was not available, so the model relied on averages. Table 2 gives the average number of cattle sold per farm for each year from 2015 to 2018. The simulation model assumed that for each of the 100,000 hypothetical farms, each of these four average numbers was equally likely.

For the simulations, we applied the simulated weight gain percentages to the base sale weight of Feeder Steers of 400kg as in Section 3.1.

The methods for calculating savings from fertiliser and reduced desilting follow those set out in the deterministic calculations. The exception is that the number of cattle per farm and the farm size are not taken as the average levels over 2015 to 2018, but rather, the value for each of the four years was assumed to be equally likely. We note that correlation likely exists between the average number of cattle on the farm, the number of cattle sold, and the size of the farm. Therefore, for a simulated farm where, for example, cattle sold per farm was taken as the 2016 average, the number of cattle and size of farm was also taken to be the 2016 average.

### 4.4 Costs

For the simulations, financial costs per dam of fencing and associated maintenance, hardened water point installation and maintenance, and vegetation, were kept fixed at the amounts set out in Section 3 above. We incorporated variation in costs only through differences in the number of farm dams, where it is assumed that one dam was constructed for every 40 hectares, and farm size varies according to the different average levels over 2015 to 2018 given in Table 2.

## 5. Results

### 5.1 Deterministic analysis

Assuming that cattle will on average gain an additional 11% in weight by switching from water sourced from dams in poor condition to dams in good condition, we found that the economic benefits outweigh the economic costs in both NSW and Victoria (Table 3). Over a 50-year period and at a real discount rate of 7% per annum, the Benefit-Cost Ratio (BCR) was calculated to be 1.5 for farms in the New South Wales (NSW) Local Government Areas in Table 2 where annual rainfall exceeds 600mm. Analyses based on Victorian farms, with their higher stocking rates, yielded a BCR of 3.0. Combining the deterministic Net Present Values (NPV) calculated per average farm in Table 3 with the mean number of farms (Table 2) resulted in an estimated NPV of $85m in 2019 prices for the farms in NSW, and $519m for Victoria. Other farms in non-marginal cattle country in NSW and Victoria would yield NPV values to varying extents, so that the overall economic gain of $604m may be considered to be a lower bound estimate.

Discounted break-even analysis was undertaken to determine the break-even point in weight gain necessary for benefits to exceed costs over the 50-year period. Our analysis indicated that cattle on farms in NSW would on average need to experience at least a 6.5% per annum higher weight gain due to switching to renovated dams to break even in present value terms. In comparison, Victorian farms would need only about a 1.8% per annum increase in weight gain to break even (Appendix II in S1 File). It is noteworthy that the percentage increases in weight gain needed to break even are very low compared to the average additional weight gain of 11% derived from the research studies available.

### 5.2 Monte-Carlo simulations

**Fig 2:**
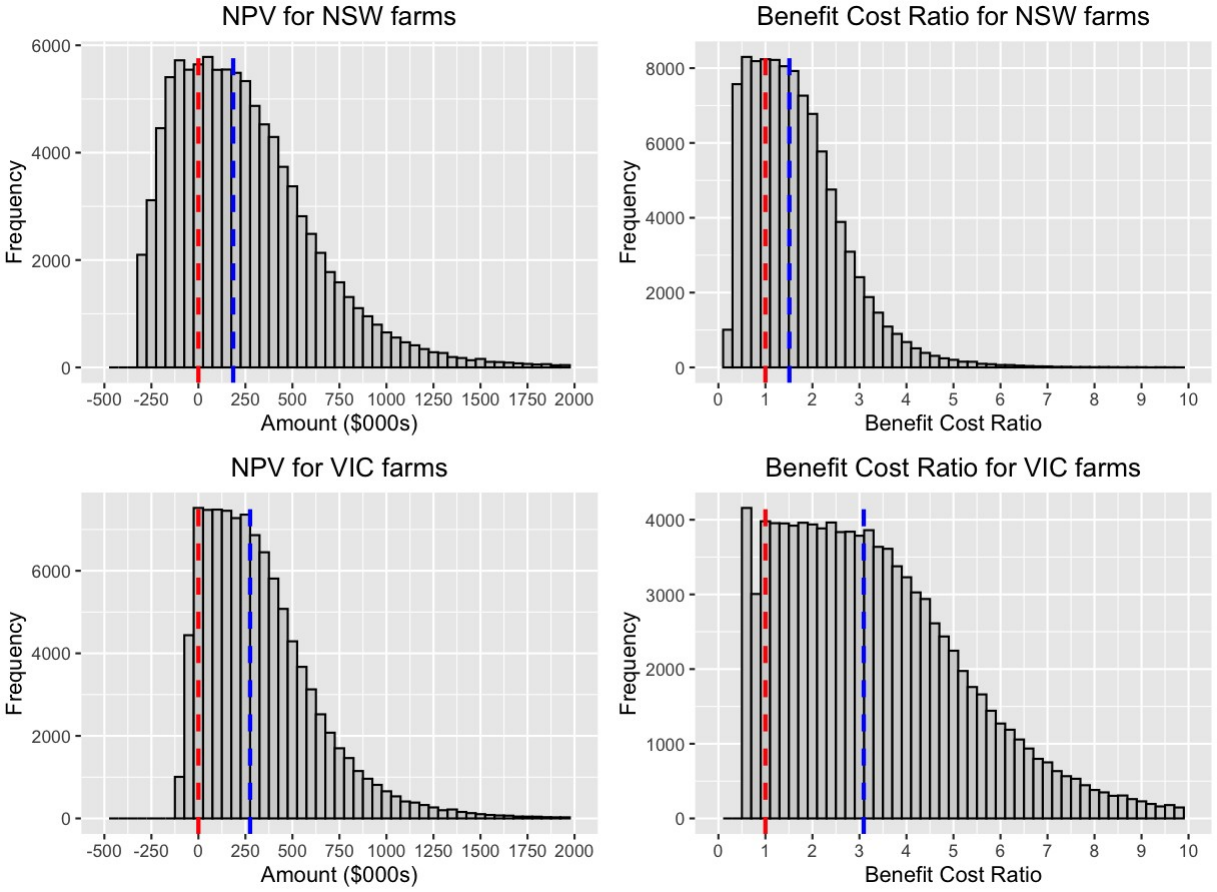
Net Present Value (NPV) and Benefit Cost Ratio (BCR) of renovating dams on beef cattle farms in NSW and Victoria. The blue dashed line is the median. In the NPV plots, the red dashed line marks the point where the NPV equals 0. In the BCR plots, the red dashed line marks the point where PV of benefits equals PV of costs.

Simulation results, which are included because of considerable uncertainty in the percentage weight gain, are given below (See Fig 2). A consequence of utilising average farm sizes and numbers of cattle when simulating benefits and costs is that the results presented below should not be taken to represent the full variation in NPV across individual farms. We did not incorporate variability in farm size, number of cattle, and number of dams in the simulations beyond the variation in the average of these measures over the years 2015 to 2018. The variation in the simulations arises predominantly because of uncertainty in weight gain and cattle prices.

We present the results of the simulation analysis in Table 4. Using a uniform probability distribution for additional weight gain, we found that the interquartile range for the Benefit-Cost Ratio was 0.9 to 2.2 for NSW and 1.8 to 4.6 for Victoria.

**Table 4:** Results of simulations of Net Present Value (NPV) and Benefit Cost Ratio per farm. NPVs are in $000s.

		NSW	Victoria
Net Present Value ($000s)	25th percentile	-37	105
	Median	185	275
	75th percentile	445	483
Benefit Cost Ratio	25th percentile	0.90	1.81
	Median	1.51	3.10
	75th percentile	2.22	4.59
Probability Benefit > Costs		70.9%	90.8%

The results of our cost-benefit analysis in Tables 3 and 4 indicate high Benefit-Cost Ratios that vary between NSW and Victoria predominantly due to different densities of cattle per hectare. Our simulations indicated a high probability that benefits would exceed costs. However, both the deterministic and stochastic results are qualified due to the sparse research on water quality and cattle weight gain that was used to inform assumptions on percentage weight gain. Specifically, the choice of 11% as an estimate of weight gain is subject to considerable uncertainty, and the simulation results rely on the untested assumption that a uniform distribution for additional weight gain with a range of 0% and 23% is appropriate in the Australian environment.

## 6. Discussion

Access to water is a key component of livestock production. The availability of good quality drinking water may, in turn, promote livestock weight gain relative to consumption of poor quality water. However, there are costs associated with improving the condition of farm dams and improving water quality. We compared the economic benefit of additional weight gain in cattle with the resource costs of renovating dams. Our analyses suggested that cattle on farms in NSW and Victoria would need to experience additional weight gain from switching to clean water of at least 6.5% and 1.8% per annum respectively, to break even in present value terms. In addition, we calculated that the average per-farm Benefit-Cost Ratios based on deterministic assumptions was 1.5 for New South Wales (NSW) and 3.0 for Victoria in areas where rainfall exceeded 600mm annually. Our results indicate that there may be substantial net economic benefits from renovating dams in poor condition.

### 6.1 Limitations of, and assumptions underpinning the study

In the absence of detailed empirical data on weight gain under Australian conditions, our modelling results are necessarily indicative. Substantial further work is required in Australia to more accurately quantify expected weight gains in cattle (and also sheep) from renovating farm dams and improving the quality of the water they are consuming. Attention should also be given to the question why so many Australian farmers persist in relying on unfenced, contaminated dams if economic gains are to be made from cleaner water. By comparison, significant efforts and investments are commonly made to improve productivity through various other means such as pasture improvement, parasite control, and genetic variation.

While the results of this study are positive, several assumptions underpin our analysis. First, there are no publicly available Australian experimental data about the expected additional weight gain of beef cattle consuming clean water, rather than water from dams in poor condition. Whether the results of the small number of Northern Hemisphere studies are valid under Australian conditions remains unclear. Second, cattle may hold different preferences relative to humans for clean versus poor-quality water. Crawford and Cole [32, pp. 4–5] found no significant difference in intakes of dam and well water. Willms, Kenzie [8] noted that steers drank for longer periods from a direct access dam than at troughs. However, actual water consumption at unfenced dams could not be measured, and it remains unclear if the same quantity was consumed from dams in poor condition, albeit more slowly. As Beede [1, p. 5–6] noted, cattle may have normal water intake rates, even if no clean water is available. A third limitation of our study was that we modelled an average number of cows sold annually per farm (Table 2) and the available data excluded farms with herds of about 100 cows or less. Fourth, we used conservative estimates for the establishment of fencing and hardened watering points to ensure robust estimation but we did not include costs of excavating new dams in our analysis. Fifth, if improved water quality results in greater feed intake, stocking rates will need to be reduced or farmers will face the additional costs of increased feed, thus reducing benefit-cost ratios.

Finally, we excluded potential positive externalities, including increased biodiversity [4, 24], or changes in greenhouse emissions [6] and mental health effects for farmers. These were not included due to lack of information. Stated preference methods could be used to estimate values that include these factors [see for example, 33].

## 7. Conclusions

We compared the economic benefit of additional weight gain in cattle with the resource costs of renovating dams. We found there was a strong likelihood of substantial net economic benefit from renovating dams in poor condition to improve water quality. Indeed, Monte Carlo simulations indicated that the probability of per-farm benefits exceeding costs was greater than 70%. Our analysis therefore suggest that incentive schemes to improve livestock production in agricultural landscapes could be focussed around renovating farms dams. Such interventions are also likely to have biodiversity benefits as well as reduce greenhouse gas emissions from the farming sector. A major area of uncertainty in our analyses is the level of weight gain in cattle associated with the consumption of improved water quality from renovated farm dams. On this basis, we suggest that the next important step is the establishment of experiments to assess the impact of improved water quality on livestock weight gain under Australian conditions.

## Supporting information

S1 File Increased livestock weight gain from improved water quality in farm dams: A cost-benefit analysis. (DOCX)

## References

[1] Beede DK. Evaluation of water quality and nutrition for dairy cattle. High Plains Dairy Conference; Albuquerque, New Mexico2006.

[2] Brew MN, Myer RO, Hersom MJ, Carter JN, Elzo MA, Hansen GR, et al. Water intake and factors affecting water intake of growing beef cattle. Livestock Science. 2011;140:297–300.

[3] Reyne M, Nolan M, McGuiggan H, Aubry A, Emmerson M, Marnell F, et al. Artificial agri-environment scheme ponds do not replicate natural environments despite higher aquatic and terrestrial invertebrate richness and abundance. Journal of Applied Ecology. 2020. doi: 10.1111/1365-2664.13738

[4] Samways MJ, Deacon C, Kietzka GJ, Pryke JS, Vorster C, Simaika JP. Value of artificial ponds for aquatic insects in drought-prone southern Africa: a review. Biodivers Conserv. 2020;29:3131–50.

[5] Srikanthan R, Barua S, Hafeez M. Estimating Volume of Water Harvested by Farm Dams in Murray-Darling Basin. 21st International Congress on Modelling and Simulation; Gold Coast, Australia2015. p. 2290–6.

[6] Ollivier QR, Maher DT, Pitfield C, Macreadie PI. Punching above their weight: Large release of greenhouse gases from small agricultural dams. Global Change Biology. 2018;25:721–32. doi: 10.1111/gcb.14477 30457192

[7] Malerba ME, Wright N, Macreadie PI. A Continental-Scale Assessment of Density, Size, Distribution and Historical Trends of Farm Dams Using Deep Learning Convolutional Neural Networks. Remote Sensing. 2021;13:319.

[8] Willms WD, Kenzie OR, McAllister TA, Colwell D, Viera D, Wilmshurst JF, et al. Effects of water quality on cattle performance. Journal of Range Management. 2002;55:452–60.

[9] Lardner HA, Kirychuk BD, Braul L, Willms WD, Yarotski J. The effect of water quality on cattle performance on pasture. Australian Journal of Agricultural Research. 2005;56:97–104.

[10] Crawford RJ, Cole E, Carpenter J. Effect of water source and quality on water intake and performance of steers grazing tall fescue. Mount Vernon, Missouri: Missouri Agricultural Experiment Station, University of Missouri-Columbia, 1997.

[11] Westgate MJ, Crane M, Scheele BC, Crane C, O’Malley C, Smith D, et al. Fencing farm dams increases vegetation cover, water quality and macroinvertebrate biodiversity. Agriculture Ecosystems and Environment. 2021;in review. 10.1002/ece3.8636 PMC8928867 35342565

[12] Cunningham R, Lindenmayer DB. Approaches to landscape scale inference and design issues. Curr Landsc Ecol Rep. 2016;2:42–50.

[13] Australian and New Zealand Environment and Conservation Council. Australian and New Zealand Guidelines for Fresh and Marine Water Quality. Canberra, Australia: Australian and New Zealand Environment and Conservation Council, 2000.

[14] Trueman KF, Clague DC. Sodium chloride poisoning in cattle. Australian Veterinary Journal. 1978;54:89–91. doi: 10.1111/j.1751-0813.1978.tb00356.x 655985

[15] Lardy G, Stoltenow C, Johnson R. Livestock and water. Fargo, North Dakota: North Dakota State University Extension Service, 2008.

[16] Murphy GM, Plasto AW. Liveweight response following sodium chloride supplementation of beef cows and their calves grazing native pasture. Australian Journal of Experimental Agriculture and Animal Husbandry. 1973;13:369–74.

[17] Wilkes J, Cowley F, Hegarty R, Jewell M. Survey of Australian feedlot drinking water quality. North Sydney, Australia: Meat and Livestock Australia, 2019.

[18] Arthur PF, Archer JA, Herd RM. Feed intake and efficiency in beef cattle: overview of recent Australian research and challenges for the future. Australian Journal of Experimental Agriculture. 2004;44:361–9.

[19] Bennett D, Morley FHW, Clark KW, Dudzinski ML. The effect of grazing cattle and sheep together. Australian Journal of Experimental Agriculture and Animal Husbandry. 1970;10:694–709.

[20] Utley PR, Bradley NW, Boling JA. Effect of restricted water intake on feed intake, nutrient digestibility and nitrogen metabolism in steers. Journal of Animal Science. 1970;31:130–5. doi: 10.2527/jas1970.311130x 5451024

[21] Lofgreen GP, Givens RL, Morrison SR, Bond TE. Effect of drinking water temperature on beef cattle performance. Journal of Animal Science. 1975;40:223–9.

[22] Wales WJ, Moran JB, Harris RW. A comparison of growth rates and carcass quality of steers receiving maize silage as a supplement to annual pasture or as a component of a feedlot ration. Australian Journal of Experimental Agriculture. 1998;38:1–6.

[23] Hazell D, Hero JM, Lindenmayer DB, Cunningham RB. A comparison of constructed and natural habitat for frog conservation in an Australian agricultural landscape. Biological Conservation. 2004;119(1):61–71.

[24] Hamilton AJ, Conort C, Aurore Bueno A, Murray CG, Grove JR. Waterbird use of farm dams in south-eastern Australia: abundance and influence of biophysical and landscape characteristics. Avian Research. 2017;8. doi: 10.1186/s40657-016-0058-x

[25] Dobes L, Leung J, Argyrous G. Social Cost-Benefit Analysis in Australia and New Zealand. The state of current practice and what needs to be done. Canberra, Australia: ANU Press; 2016.

[26] Meat and Livestock Australia. Saleyard cattle indicators—NSW [and Victoria]. Quarterly 2020 [accessed 20 March 2020]. https://www.mla.com.au/prices-markets/market-reports-prices/.

[27] Fulhage CD, Pfost DL. Fertilizer nutrients in dairy manure. Missouri, USA: University of Missouri Extension, 1993.

[28] Associates Sillar. Cost benefit study of riparian restoration on the Mary River. Queensland: Sillar Associates, 1999.

[29] Evidentiary Pty Ltd. What are the benefits to landholders of adopting riparian works? A summary of evidence and technical information.. Melbourne, Victoria: Department of Environment, Land, Water and Planning, 2016.

[30] Crossman S, Li O. Surface Hydrology Points (Regional) Canberra, Australia: Geoscience Australia; 2015 [accessed May, June 2020]. http://pid.geoscience.gov.au/dataset/ga/83132.

[31] Ede F. Riparian works evaluation project: final report. Melbourne, Australia: Department of Primary Industries, 2011.

[32] Crawford RJ, Cole E. Effect of water source and quality on water intake and performance of cows and calves grazing tall fescue. University of Missouri-Columbia, Mount Vernon, Missouri: Missouri Agricultural Experiment Station, 1999.

[33] Bateman IJ, Carson RT, Day B, Hanemann M, Hanley N, Hett T, et al. Economic valuation with stated preference techniques: a manual. Cheltenham, England: Edward Elgar; 2002.

