# Supplementary material for: Increased livestock weight gain from improved water quality in farm dams: A cost-benefit analysis
Source: PLoS One. 2021 Aug 16;16(8):e0256089. doi: 10.1371/journal.pone.0256089 (PMC8366965; doi:10.1371/journal.pone.0256089)
Supplement: S1 File — (DOCX) [file pone.0256089.s001.docx]

**S1 File: Increased livestock weight gain from improved water quality in farm dams: a cost-benefit analysis**

**Appendix I: Practitioners, academics and officials consulted.**

Malcolm Ballard, Foggy’s Spraying and Rural Contracting, Adjungbilly.

Philip Batterham, Director, Centre for Mental Health Research, ANU.

Greg Bekker, Livestock Extension Officer, Agriculture Victoria, Rutherglen.

Jeff Bennett, Emeritus Professor, Crawford School of Public Policy, ANU.

John Broster, Senior Technical Officer, Graham Research Centre, Charles Sturt University, Wagga Wagga.

John Casnave, Gundagai Earthworks, Gundagai.

Adam Cheetham, Senior Market Analyst – Market Information, Meat & Livestock Australia.

Clare Crane, Biodiversity Field Officer, Sustainable Farms, ANU.

Mason Crane, Senior Research and Extension Officer, Sustainable Farms, ANU.

Amber Croft, Tamara Harris, Kathie LeBusque: Sustainable Farms Engagement and Events Coordinators.

Paul Cusack, Consultant, Cattle Veterinarian and Ruminant Nutritionist, Cowra, and Adjunct Professor, Charles Sturt University.

Albert van Dijk, Professor Water Science and Management, Fenner School, ANU.

Madeline Dunk, Research Assistant, ANU.

Dan Florance, Research and Extension Officer, Sustainable Farms, ANU.

Gervaise Gaunt, Regional Manager, Northern Meat and Wool, Agriculture Victoria, Rutherglen.

Michael Gooden, Riverina Local Land Services, Regional Agricultural Landcare Facilitator, Wagga Wagga.

Eve Hall, District Veterinarian, Murray Local Land Services, NSW Department of Primary Industries, Holbrook.

Graeme Harris, farmer, Coolac.

Shahid Khalfan, Associate Lecturer, Ruminant Health and Production, Charles Sturt University, Wagga Wagga.

Kylie Macreadie, Team Leader (Meat and Wool), Agriculture Victoria, Rutherglen.

Craig McGrath, Craig McGrath & Son, Fencing Contractors, Gundagai.

Shawn McGrath, School of Animal and Veterinary Sciences, CSU, Wagga Wagga.

Bill Malcolm, Associate Professor, Department of Agriculture and Food Sciences, University of Melbourne.

Jock Mason, Mason’s Bulldozing, Adjungbilly.

Chris Mirams, Director Meat and Livestock Australia, and Director Sheep Producers Australia.

John Piltz, Livestock Research Officer, NSW Department of Primary Industries and Charles Sturt University, Wagga Wagga.

Gordon Refshauge, Livestock Research Officer, NSW Department of Primary Industries, Cowra.

David Smith, Research and Extension Officer, Sustainable Farms, ANU.

Vernon Topp, Dale Ashton, James Frilay, Australian Bureau of Agricultural and Resource Economics and Sciences, Australian Government, Canberra.

David Trengove, Senior Livestock Production Advisor, Elders Pty Ltd, Cowra.

Dennis Watson, Irrigation Officer, Agriculture Victoria, Rutherglen.

**Appendix II: Approximate per farm break-even Net Present Value (NPV) of renovating dams on beef cattle farms over a 50-year period, assuming an annual weight gain of 6.5% for NSW and 1.8% for Victoria. ($’000)**

|  |  |  |
| --- | --- | --- |
| Item | NSW: per farm | Victoria: per farm |
|  | PV($2019) | PV($2019) |
| ***Benefits*** |  |  |
| Value of additional weight gain | 248,386 | 53,291 |
| Fertiliser saving | 49,995 | 59,242 |
| Saving due to reduced frequency of desilting dams | 73,981 | 27,507 |
| Present Value ($2019) of benefits | 372,361 | 140,040 |
|  |  |  |
| ***Costs*** |  |  |
| Construction of dam fence | 203,944 | 75,829 |
| Construction of hardened watering point | 113,538 | 42,215 |
| Planting vegetation | 13,943 | 5,184 |
| Dam fence maintenance | 13,023 | 4,842 |
| Hardened watering point maintenance | 26,803 | 9,966 |
| Present Value ($2019) of costs | 371,250 | 138,035 |
|  |  |  |
| Net Present Value ($2019) | 1,111 | 2,005 |
| Benefit Cost Ratio | 1.0 | 1.0 |
| *Notes*: Cost and Benefit data used were identical to those in Table 3 in the text. However, iterative numerical manipulation of the annual percentage weight gain was used to drive the Net Present Value (NPV) as close to zero as possible without generating a negative NPV value. The percentage weight gain figure was 6.5 for NSW farms and 1.8% for Victorian farms. An assumed annual weight gain of 6.4% yields an NPV = -2,710 and 1.7% for Victoria would yield an NPV = -956. | | |
